# Diabetic Nephropathy: Proteinuria, Inflammation, and Fibrosis

**DOI:** 10.1155/2016/5241549

**Published:** 2016-01-06

**Authors:** Shirong Zheng, David W. Powell, Feng Zheng, Phillip Kantharidis, Luigi Gnudi

**Affiliations:** ^1^Department of Cardiovascular and Thoracic Surgery, Cardiovascular Innovation Institute, University of Louisville, Louisville, KY 40202, USA; ^2^Department of Medicine and Division of Nephrology and Hypertension, School of Medicine, University of Louisville, Louisville, KY 40202, USA; ^3^Department of Nephrology, The Second Hospital and Advanced Institute for Medical Sciences, Dalian Medical University, Dalian 116044, China; ^4^JDRF Danielle Alberti Memorial Centre for Diabetes Complications, Baker IDI Heart and Diabetes Institute, Melbourne, VIC 3004, Australia; ^5^Cardiovascular Division, School of Life Science & Medicine, King's College London, Department of Diabetes and Endocrinology, Guy's Hospital, London SE1 9RT, UK

Diabetic nephropathy (DN) is a serious complication of diabetes; it initially manifests with microalbuminuria and progresses towards end-stage renal failure. Sustained diabetes-related metabolic and haemodynamic perturbations can induce subclinical low-grade renal inflammation and drive kidney from repair response to damage process, eventually to renal fibrosis. In this special issue, we include articles regarding inflammation, Chinese herbs, and systems biology to present up-to-date information on immune cells, chemokine receptor, and biomarkers in DN, displaying combined therapy in treatment of DN and highlighting the effective approach in exploring genetic susceptibility of DN.

*(1) Inflammation*. Despite the broad themes covered by this special issue, all articles focus on a common theme: immune cells and inflammation. Hyperglycemia and oxidative stress, as well as albuminuria per se, can lead the immune and inflammatory cells to infiltrate into kidney and release proinflammatory cytokines. This inflammatory “repair process” reverts to and manifests as a “chronic unfavorable process” that eventually leads to the disease phenotype (renal fibrosis). The review article by Z. Zheng and F. Zheng summarizes the role of immune cells and inflammation in DN, highlighting the contribution of APC cells, T-helper cells, and tubular epithelial cells to the inflammation. S. Zheng et al. reported the renal expression of decoy chemokine receptor ACKR2 in DN patients and renal protection in diabetic mice with ACKR2 gene knockout, revealing the unexpected negative role of ACKR2 in diabetic kidney disease. Association of haemostatic and inflammatory biomarkers with nephropathy in type I diabetic patients is shown by C. P. Domingueti et al., indicating potential therapeutic targets for DN.

*(2) Chinese Medicine*. Herbs are major form of therapy in traditional Chinese medicine. Their value has been illustrated by the discovery of artemisinin [1], a drug saving millions of lives from malaria, derived from an active ingredient of Chinese herb. Chinese medicine is part of the treatment used against DN in China. In review by G. Sun et al., over 20 recipes of herb medicine and 30 single herbs or monomers are summarized. These therapies have showed efficacy at improving albuminuria and inflammation in diabetic patients. Ongoing research programs focus on identifying the effective component(s) contained in the most promising herbs with the ultimate aim of developing safe and novel compounds for the treatment of DN.

*(3) Systems Biology*. As defined by NIH, systems biology is an approach used in biomedical research to understand the “bigger picture”—be it at the level of the organism, tissue, or cell—to reconstruct the biology from huge volumes data using computational and mathematical methods. This is in stark contrast to decades of reductionist biology, which involved taking the pieces apart in order to understand the biology [2]. As technology advances, genomics, proteomics, and metabolomics become reliable, affordable, and readily available to explore the molecular profiles of human disease. F. Conserva et al. present a systems biology overview of human DN, from genetic susceptibility to posttranscriptional and posttranslational modifications. Molecules identified by genomics, transcriptome, and epigenetic studies in area of DN await to be validated. Using proteomics approach, M. Barati et al. report the influence of acute high glucose exposure on the change in protein abundance in murine glomerular mesangial cells. These discovery-based studies shed new light and new perspectives in DN research.
